# Quantification of 1,3-olein-2-palmitin (OPO) and Palmitic Acid in *sn*-2 Position of Triacylglycerols in Human Milk by Liquid Chromatography Coupled with Mass Spectrometry

**DOI:** 10.3390/molecules24010022

**Published:** 2018-12-21

**Authors:** Francesca Giuffrida, Cynthia Marmet, Isabelle Tavazzi, Patric Fontannaz, Julien Sauser, Le Ye Lee, Frédéric Destaillats

**Affiliations:** 1 Nestlé Research, Vers-chez-les-Blanc, 1000 Lausanne 26, Switzerland; cynthia.marmet@rdls.nestle.com (C.M.); isabelle.tavazzi@rdls.nestle.com (I.T.); patric.fontannaz@rdls.nestle.com (P.F.); julien.sauser@rdls.nestle.com (J.S.); 2 The Children′s Medical Institute, National University Hospital, Singapore 637551, Singapore; le_ye_lee@nuhs.edu.sg; 3 Department of Pediatrics, Yong Loo Lin School of Medicine, National University of Singapore, Singapore 637551, Singapore; 4 Nestlé Nutrition, 1800 Vevey, Switzerland; frederic.destaillats@nestle.com

**Keywords:** 1,3-olein-2-palmitin, human milk, high resolution mass spectrometry

## Abstract

This study describes the identification and quantification of fatty acids in the *sn*-2 position of triacylglycerols (TAG) and of the most abundant TAG regioisomers in human milk by liquid chromatography coupled with high-resolution mass spectrometry (HPLC-HRMS). Over 300 individual TAG species were observed and 1,3-olein-2-palmitin (OPO) was identified as the most abundant TAG regioisomer. Validation of the HPLC-HRMS method showed repeatability and intermediate reproducibility values ranging from 3.1 to 16.6% and 4.0 to 20.7%, respectively, and accuracy ranging from 75 to 97%. Results obtained by the HPLC-HRMS method were comparable to results from the ISO 6800 method for the quantification of palmitic acid in the *sn*-2 position of TAG (81.4 and 81.8 g 100 g^−1^ total palmitic acid, respectively). Processing the data obtained with the HPLC-HRMS method is extremely time consuming and, therefore, a targeted method suitable for the quantification of OPO in human milk samples by ultra-performance (UP) LC coupled with triple quadrupole (QQQ) MS was developed and validated. OPO identification and quantification by UPLC-QQQ were based on nominal mass and a fragmentation pattern obtained by multiple reaction monitoring experiments. The method was validated in terms of accuracy and precision by analyzing different aliquots of the same human milk sample over time and comparing the results with values obtained by HPLC-HRMS. Intermediate reproducibility was <15% and trueness comparable to HPLC-HRMS. Quantification of OPO in human milk samples collected at 30, 60 and 120 days postpartum showed that OPO content varies between 333 ± 11.8 and 383 ± 18.0 mg 100mL^−1^.

## 1. Introduction

Human milk is considered the optimal form of nutrition for infants and is the main food for a healthy infant during the first 4–6 months of life [1]. The lipid fraction provides approximately 50% of the energy supplied to the infant and it is mainly composed of triacylglycerols (TAG) representing about 98% of total lipids [2,3,4]. The vast majority of fatty acids (FA) are esterified to glycerol as TAG and about 0.2–2% are found esterified to other molecules such as cholesterol, phospholipids, sphingolipids and glycosphingolipids. In TAG, FA esterified in the external positions of the glycerol are named *sn*-1(3) or alpha FA while FA esterified in the internal position are named *sn*-2 or beta FA. The FA found in milk are mainly synthetized de novo in the mammary gland or incorporated from FA supplied by the diet [5]. The synthesis of saturated FA is influenced by diet quality and it has been demonstrated that the levels of 6:0–14:0 FA in milk are increased when lactating women consume diets rich in carbohydrate [6,7,8], whereas the content of 18 carbon FA which are derived from the diet, e.g., 18:1 n-9 and 18:2 n-6, are reduced [6,7]. Among polyunsaturated FA, linoleic (LA, 18:2 n-6) and alpha linolenic acids (ALA, 18:3 n-3) are essential because they are not synthesized in the human body, and are therefore mainly obtained through the diet. They are precursors of arachidonic (20:4 n-6) and docosahexaenoic (22:6 n-3) acids, which are associated with normal brain development especially in early life [9].

Palmitic acid (PA, 16:0) is the most abundant saturated FA in human milk, its level is relatively constant and represents about 20–25% of the total milk FA irrespective of the country of origin and the diets of the mothers, exception made for vegetarians [4,8]. In human milk, over 70% of the palmitic acid content is esterified in the *sn*-2 positon of TAG [8,10] allowing greater efficiency of palmitic acid absorption and utilization in breast-fed infants when compared to infants fed with formula containing PA preferentially esterified in *sn*-1(3) position of TAG [4]. During digestion, pancreatic lipase releases preferentially the FA in the *sn*-1(3) positions of the TAG to produce FA and 2-monoacylglycerols [11]. PA esterified in the *sn*-2 position of monoacyglycerol is mostly absorbed while free PA can react with calcium to form insoluble calcium soaps [4]. A few studies have observed that the formation of calcium soaps of saturated FA might results in the production of harder stools and constipation in infants [12,13]. Therefore, the regiospecific distribution of saturated FA is important to support the optimal utilization of palmitic acid and calcium absorption in infants and an extensive review on the subject was recently published by Petit et al. [14].

TAG composition in human milk has been investigated extensively [10,15,16,17,18,19,20,21,22,23,24,25,26,27,28,29]. However, detailed information on the absolute amount of TAG species and FA in *sn*-2 positions are lacking and since *sn*-2 FA positions are maintained after absorption, the purpose of the milk TAG structure may be more sophisticated than simply avoiding PA malabsorption [18].

Methods based on thin-layer chromatography (TLC) [10] and the partial hydrolysis of TAG with pancreatic lipase [16,19,28] or with Grignard reagent [24] have been used to quantify the FA esterified in *sn*-2 position of TAG in human milk. Morera Pons et al. [23] described a nonaqueous reversed phase (NARP) chromatography method coupled with an evaporative light-scattering detector without previous TLC separation or partial hydrolysis of TAG. The retention in NARP mode is ruled by the equivalent carbon number (ECN = carbon number − 2 × double bonds). However, TAG identification based on ECN is complicated and TAG structure elucidation and quantification required the use of mass spectrometry (MS) [25]. Recently, a few NARP-MS methods, without previous TLC separation or partial hydrolysis of TAG and mainly using atmospheric pressure chemical ionization (APCI) have been employed for the TAG characterization and quantification in human milk [20,25,26,27,29].

The main objective of the present study was to explore the diversity of TAG structures in human milk by high-resolution mass spectrometry (HRMS) and optimize a method to quantify the major TAG regioisomers and FA in the *sn*-2 position of TAG focusing on PA due to its relevance for the nutrition of infants.

## 2. Results

### 2.1. High Performance Liquid Chromatography-High-Resolution Mass Spectrometry (HPLC-HRMS) Method Validation

**Figure 1:**
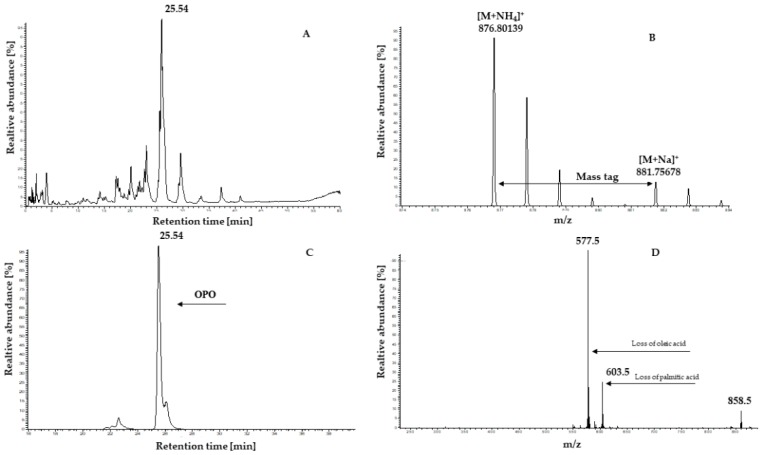
(**A**) High-resolution base peak ion chromatogram of human milk obtained in the Orbitrap. (**B**) Mass tag between ammoniated and sodiated ions that serves as an additional triggering criterion. (**C**) High-resolution ion chromatogram of ion 876 Da (1,3-olein-2-palmitin, OPO) that serves as a basis for quantification. (**D**) Averaged product ion mass spectrum of parent ion 876 Da obtained in the linear ion trap in parallel to the high-resolution chromatogram; only monoisotopic product ions are present, representing the loss of PA (603.5 *m*/*z*), oleic acid (577.5 *m*/*z*) and of one ammonia unit (858.5 *m*/*z*).

Lipid extracts were analysed and typical results obtained from human milk using the described high-performance liquid chromatography coupled with high-resolution mass spectrometry (HPLC-HRMS) approach are shown in Figure 1.

As previously reported [30], under the described conditions both ammonium and sodium adducts from TAG are produced. Therefore, if the (*i*) exact mass inclusions list, (*ii*) mass tag, i.e., difference between the ammoniated and sodiated adduct (*m*/*z* 4.95539, see Figure 1), and (*iii*) intensity threshold criteria were met, the system performed a product ion scan on the ammoniated TAG (Figure 1). The peaks in the TAG product ion spectrum were used to identify the FA residues and their intensity was used to determine the FA regioisomeric position according to the mathematical model described previously [30]. The concentration of TAG regioisomers was determined by integrating the corresponding peak areas in the HR ion chromatograms, normalizing them to the peak area of the internal standard and inserting them in a calibration curve prepared daily using the TAG species listed in Table S1 (supplementary material). The HPLC-HRMS method was validated to assess linearity, limit of quantification (LOQ), precision and trueness. In order to evaluate the linearity, six different concentrations of standard solution of TAG species (Table S1 Supplementary material) covering ranges from 0.01 to 10 μg mL^−1^ were injected over six days. Due to the lack of linearity (R^2^ < 0.8), polynomial equations were used. The LOQ was defined as the lowest concentration of the calibration curve since the analyte response was at least 5 times higher than the blank response and reproducible with a precision of at least 20% [31]. Precision results of the lowest point of the calibration curve, ranged between 1.8 and 17.8% (Table S1 Supplementary material). In order to evaluate the precision of the HPLC-HRMS method, different aliquots of the same human milk were analyzed on seven different days by two different operators (Table S2 supplementary material). Repeatability values ranged between 3.1 and 16.6% and intermediate reproducibility between 4.0 and 20.7%. According to United States Food and Drug Administration (FDA) guidelines [31] precision values lower than 15% and 20% at concentrations close to the LOQ are acceptable. The trueness was evaluated by comparing *sn*-2 PA content in human milk analyzed by the ISO 6800 [32] method and the HPLC-HRMS method. Comparable results were observed between the lipase based ISO 6800 (81.8 g 100 g^−1^ PA) and HPLC-HRMS (81.4 g 100 g^−1^ PA) methods.

### 2.2. Triacylglycerols (TAG) Regioisomers Quantification in Human Milk by HPLC-HRMS Analysis

**Figure 2:**
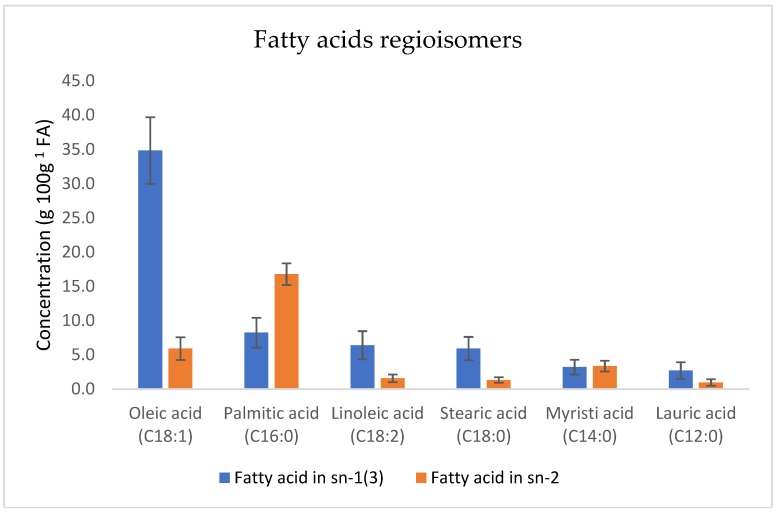
Human milk regioisomeric distribution of fatty acids (FA) in triacylglycerols (TAG), combining human milk samples collected between 0 and 120 days after delivery. Results are expressed in g 100 g^−1^ FA. Error bars represent the standard deviation with *n* = 55.

The quantification of the main fatty acids esterified in the *sn*-2 position, and the identification of the most abundant TAG regioisomer is a HPLC-HRMS heavy task and is time consuming; therefore, the analysis was performed on a small set of human milk samples (*n* = 55) collected between 0 and 120 days *postpartum*. Figure 2 shows the regioisomer distribution of the most abundant FA in human milk.

Palmitic acid (PA) (16.8 ± 1.6 g 100 g^−1^ FA) was the most abundant fatty acid esterified in the *sn-*2 position followed by oleic (5.9 ± 1.7 g 100 g^−1^ FA) and myristic (3.4 ± 0.8 g 100 g^−1^ FA) acids. When considering total PA, 65% was esterified in *sn*-2 position (Table 1).

This is in agreement with previous studies [16,17,18,19,28] reporting between 51 and 88% of *sn*-2 PA. Oleic acid (34.8 ± 4.9 g 100 g^−1^ FA) was the most abundant FA esterified in *sn-*3 position followed by PA (8.2 ± 2.2 g 100 g^−1^ FA) and LA (6.4 ± 2.1 g 100 g^−1^ FA). When considering the TAG profile of human milk (*n* = 55) sampled between 0 and 120 days *postpartum*, over 300 TAG species were identified (Table 2), with OPO (Table 3) being the most abundant (13.9 ± 2.1% of total TAG), followed by LPO (1-linolein-2-palmitin-3-olein, 6.0 ± 1.5%), OPP (1-olein-2,3-palmitin 5.7 ± 1.6%), OPS (1-olein-2-palmitin-stearin, 5.2 ± 1.4%), OOO (triolein 3.6 ± 1.5%), and POO (1-palmitin-2,3-olein 2.9 ± 1.0%). Other TAG species were present at levels lower than 3%.

### 2.3. 1,3-Olein-2-Palmitin (OPO) Quantification by Ultra-Performance Liquid Chromatography Coupled with Triple Quadrupole Mass Spectrometry (UPLC-QQQ)

HPLC-HRMS data treatment is heavy and time consuming therefore it is not suitable for the analysis of large sets of samples. In order to quantify the OPO content in a large set of samples, human milk (*n* = 143) collected 30, 60 and 120 days postpartum was analyzed by UPLC-QQQ. Lipids were extracted as described in paragraph 5.3 and the OPO ammoniated adduct (876.8 *m*/*z*) was monitored for the loss of O producing the fragment at 577.5 *m*/*z* and the loss of PA producing the fragment at 603.5 *m*/*z.* The OPO concentration was determined by integrating fragment peak area in MS/MS chromatograms, normalizing them to the peak area of the internal standard and substituting into the experimentally daily determined calibration curve using an OPO standard. The UPLC-QQQ method was validated to assess linearity, LOQ, precision and trueness. In order to evaluate the linearity, six different concentrations of OPO standard solutions covering ranges from 0.01 to 7.5 μg mL^−1^ were injected over six days. Due to the lack of linearity (R^2^ = 0.98,) a polynomial equation was used. The LOQ was defined as the lowest concentration of the calibration curve because the analyte response was at least 5 times higher than the blank response and reproducible with a precision of 7%. In order to evaluate the precision of UPLC-QQQ method, different aliquots of the same human milk were analyzed over seven days by two different operators. Results showed good intermediate reproducibility with a value of 14% (Table 4).

According to FDA guidelines [31] precision values lower than 15% and 20% at concentrations close to the LOQ are acceptable. The trueness was evaluated by comparing OPO concentrations obtained in different samples of human milk analyzed by UPLC-QQQ to the values obtained by HPLC-HRMS method (Table 4).

In order to compare OPO concentrations (*n* = 36) measured by UPLC-QQQ and HPLC-HRMS, two types of bias (systematic and proportional) were checked and none was detected.

Finally, human milk samples (*n* = 143) collected 30, 60 and 120 days postpartum were analyzed for their content of OPO (Table 5).

The OPO content in human milk sampled at 30, 60 and 120 days postpartum was 333 ± 11.8, 337 ± 17.0 and 383 ± 18.0 mg 100 mL^−1^, respectively, and it did not change significantly during the lactation period.

## 3. Discussion

In human milk, TAG synthesis involves specific esterification of FA at the *sn*-1,3 and *sn*-2 positions, resulting in different TAG structures compared to the ones found in common dietary fats and oils. During TAG digestion, pancreatic lipase preferentially hydrolyzes FA at the *sn*-1,3 position releasing free FA and *sn*-2-monoacylglycerol [11]. In the presence of bile salts, *sn*-2-monoacylglycerol forms mixed micelles which are absorbed by passive diffusion and resynthesized into chylomicron TAG in the erythrocytes [33]. When palmitic acid is in the *sn*-2 position, it is well absorbed as 2-monoacylglycerol, but when located in the *sn*-1,3 positions, it is released as free FA and forms insoluble calcium soaps lowering the efficiency of fatty acid and calcium absorption and increasing the stool hardness [12,13].

In this study, we developed and validated HPLC-HRMS and UPLC-QQQ analytical methods for the quantification of FA in *sn*-2 and OPO, respectively, in lipids extracted from human milk. We showed that both analytical methods are precise (CV(r) and CV(iR) <15% and 20% for concentrations close to LOQ) and true when compared to the reference method, i.e., lipase-based ISO 6800. The ISO reference method can be used to quantitatively determine the relative FA distribution in TAG species but does not provide insight on the TAG molecular profile. Using the developed method, we confirmed that PA is preferentially esterified in the *sn*-2 position (16.8 ± 1.6 g 100 g^−1^ FA and 65 g 100 g^−1^ of overall PA) and oleic acid in *sn*-1,3 (34.8 ± 4.9 g 100 g^−1^ FA). Conversely, in lipids used to manufacture infant formula, PA is mostly esterified in the *sn*-1,3 positions, as is the case for palm oil for example, which is one of the major sources of PA in infant formula. When considering total palmitic acid, only 9% is esterified in the *sn*-2 position, therefore less than in human milk, which may explain why formula-fed infants have lower absorption of both saturated fatty acids and Ca and firmer stools than breast-fed infants [14].

We showed that OPO is the most abundant TAG in human milk and that its concentration ranged between 333 ± 11.8 and 383 ± 18.0 mg 100mL^−1^, representing about 15 g 100 g^−1^ of TAG in agreement with values (14.1–26.3 g 100 g^−1^ of TAG) previously reported by Zou et al. [29]. To our knowledge, this is the first time that absolute concentration of OPO in human milk has been reported during the lactation period.

## 4. Conclusions

In this study two procedures based on liquid chromatography coupled with mass spectrometry have been established and validated. An HPLC-HRMS procedure was used to identify over 300 individual TAG species and to determine the major fatty acid distribution in *sn*-2 and *sn*-1(3) positions of TAG in human milk; the UPLC-QQQ procedure was used for the absolute quantification of OPO in a large set of human milk samples during the lactation period. Future work should investigate the fatty acid position of TAG and OPO content in human milk of mothers delivering pre-terms and infants small for their gestational age.

## 5. Materials and Methods

### 5.1. Human Milk Collection from Mothers

The protocol and collection of human milk was reviewed and approved by the local National healthcare group Domain Specific Ethics Board of Singapore. The study was registered in ClinicalTrial.gov (NCT01805011) and took place at National University of Singapore. Volunteer mothers, who were non smokers and apparently healthy who delivered a term infant (*n* = 50; 31.1 ± 3.1 years old) provided breast milk samples of approximately 30 mL each at 30, 60 and 120 days postpartum. Samples were collected after full expression from one breast using a milk pump and while the baby was fed on the other breast to produce a satisfactory let-down in the absence of suckling response. We made all efforts to collect complete feed that included fore-milk, mid-milk and hind-milk as a representation of one feed and to avoid within-feed variation of lipid and other nutrient contents. Approximately 30 mL aliquot was separated into two conical 15 mL polypropylene tubes for this study and the rest was fed to the infant. Samples collected for research were stored at −80 °C and shipped on dry ice for analyses to Nestlé Research, Vers-chez les-Blanc, Switzerland.

### 5.2. Chemicals

HPLC-grade water, ammonium-formate, methanol, and isopropanol were obtained from Chemie Brunschwig AG, Basel, Switzerland. LC-grade sodium-formate, acetone, NH_4_ 25% diethyl ether, petroleum ether, *n*-hexane were purchased from Sigma-Aldrich, Buchs, Switzerland. Stable isotope labeled 1,3(d5)-dipalmitoyl-2-stearoyl-glycerol was obtained from Avanti Polar Lipids Inc. (Alabaster, AL, USA). All other TAG standards were purchased from Larodan/Chemie Brunschwig (AG, Basel, Switzerland).

### 5.3. Lipid Extraction from Human Milk

The Röse-Gottlieb (ISO 1211) [34] method was used as the reference method for the quantification of total lipids. In order to decrease the sample size needed for analysis, the method was slightly modified: sample size was decreased to 100 μL and solvent volumes reduced in order to maintain the same proportions sample/solvent of the reference method. Briefly, human milk (100 μL) was mixed with 2 mL of warm water (about 40 °C) and put in the ultrasound bath for 10 min at 40 °C. After sonication, an aliquot (100 μL) of the sample solution was mixed with 250 μL of internal standard solution (1,3(d5)-dipalmitoyl-2-stearoyl-glycerol 0.4 μmol L^−1^) and warm water (2.9 mL). In order to precipitate protein and separate carbohydrates, NH_4_ 25% solution (500 μL) and ethanol (2 mL) were added and finally for extracting lipids, diethyl (5 mL) and petroleum (5 mL) ether were added. After centrifugation, the organic upper phase was transferred into another tube. The residue was extracted a second time by adding ethanol (1 mL), diethyl ether (3 mL) and petroleum ether (3 mL), centrifuged (10 min at 2500 rpm), and the upper organic phase combined with the previous one. In order to maximize the lipid extraction, the residue was extracted a third time by adding diethyl ether (3 mL) and petroleum ether (3 mL), centrifuged (10 min at 2500 rpm), and the upper organic phase combined with the previous ones. The solvent was evaporated under a gentle N_2_ stream. Before analysis, the residue was diluted in acetone/methanol solution (4/1).

### 5.4. TAG Regioisomers Quantification in Humans by HPLC-HRMS Analysis

The analysis was performed as previously described [31]. Briefly, chromatography separation was achieved with a Dionex Ultimate 3000 (ThermoFisher Scientific, Bremen, Germany) equipped with an Agilent Poroshell 120 EC-C18 column (150 × 2.1 (i.d.) mm, 2.7 µm: Agilent Technologies, Santa Clara, CA, USA). Solvent A consisted of *n*-hexane and isopropanol (1:1, *v*:*v*). Solvent B was 10 mmol L^−1^ ammonium-formate solubilized in methanol. An LTQ-Orbitrap Elite hybrid mass spectrometer (ThermoFisher Scientific) was used for identification of TAG regioisomers. Electrospray ionization in positive ion mode was applied to form ions at 300 °C nebulizer temperature and 4.5 kV capillary voltage. Nebulizer and auxiliary gases were nitrogen at 40 and 20 units, respectively. Capillary temperature was 275 °C, the S-Lens RF level was adjusted to 60% and accumulation time was 300 ms. The Orbitrap was operated at 30,000 resolution in *m*/*z* between 200 and 1500. Data-dependent events were produced according to an inclusion list with the accurate masses of ammoniated TAG and applying parent mass width criteria of ±5 ppm. The inclusion list was created by calculating the accurate mass for TAG obtained by the combination of the most abundant FA in human milk (Table S3 Supplementary material). The mass tag of *m*/*z* 4.95540 between ammoniated and sodiated adducts (Figure 1) was used, but only the ammoniated adducts were fragmented.

Maximum injection time was 200 ms, isolation width was *m*/*z* of 3, normalized collision energy 30%, activation Q value was 0.250, activation time was 30 ms. Five fragmentation events per one scan cycle were triggered. Finally, the dynamic exclusion parameters were: repeat count 1; repeat duration 0 s; exclusion list size 45; exclusion duration 2.5 s; exclusion mass width ±5 ppm

### 5.5. HPLC-HRMS Method Validation

HPLC-HRMS method validation was performed to assess the linearity, limit of quantification (LOQ), accuracy and precision.

*Linearity; limit of quantification (LOQ).* The linearity of the method was assessed by analyzing six different concentrations of standard solutions of TAG species (Table S1 Supplementary material) covering ranges from 0.01 to 10 μg mL^−1^.

According to the FDA [31], the lowest concentration of the calibration curve should be accepted as the LOQ if the analyte response is at least 5 times higher than the blank response and if this response is reproducible with a precision of at least 20%.

*Accuracy*. In order to evaluate the accuracy of the method, comparison with ISO 6800 [32] method was performed for the quantification of PA in the *sn*-2 position. The ISO 6800 method was performed by an external laboratory (ITERG, Bordeaux, France).

*Precision*. The precision of the method was evaluated by calculating the repeatability (r) and the intermediate reproducibility (iR).

### 5.6. OPO Quantification in Humans by UPLC-MS/MS Analysis

Chromatographic separation of OPO was achieved with a Shimadzu Nexera LC 30 (ThermoFisher Scientific, Bremen, Germany) equipped with an Agilent Poroshell 120 EC-C18 column (150 × 2.1 (i.d.) mm, 2.7 µm: Agilent Technologies, Santa Clara, CA, USA). Solvent A consisted of *n*-hexane and isopropanol (1:1, *v*:*v*). Solvent B was 10 mmol L^−1^ ammonium-formate solubilized in methanol. An ABSciex 5500 triple quadrupole mass spectrometer (Applied biosystems/MSD Sciex, Ontario, ON, Canada) equipped with an electrospray ionization (ESI) ion source was used for identification of OPO.

The ESI mass spectra (60–900 *m*/*z*) were recorded in the positive ion mode under the following conditions: ion spray voltage 5.5 kV; curtain gas 25 psi; collision gas 9; declustering potential 160 V; Collision Cell Exit Potential 8 V; source temperature was set to 100 °C; GS1 40; GS2 30. Resolution was adjusted to approximately 0.7 full-width-at half-maximum. OPO (876.8 *m*/*z*) was monitored by the loss of oleic acid leading to the fragment at 577.5 *m*/*z* and the loss of palmitic acid leading to the fragment at 603.5 *m*/*z.* For all transitions, a dwell time of 0.3 ms and a span of 0.1 m/z were used.

Quantification was performed using a calibration curve. Stock solutions of OPO standards were prepared in acetone/methanol (4/1) to obtain at least six concentration levels covering range from 0.1 to 7.5 μg mL^−1^. The method validation was performed by analyzing minimum sixteen aliquots of the same human milk over at least 6 days by one operator by UPLC-MS/MS and HPLC-HRMS.

## Figures and Tables

**Table 1:** Palmitic acid (PA) regioisomer distribution in triacylglycerol from human milk (*n* = 55). Results are expressed as g 100 g^−1^ of total palmitic acid.

PA	Mean (g 100 g^−1^ PA)	SD (r) (g 100 g^−1^ PA)	Min (g 100 g^−1^ PA)	Max (g 100 g^−1^ PA)
*sn*-2	67.81	4.65	60.8	76.7
*sn*-1(3)	32.19	4.65	23.3	39.2

**Table 2:** Triacylglycerol species identified in human milk (*n* = 55).

Triacylglycerol Species			
18:1-16:0-18:1	12:0-18:1-14:0	16:0-18:3-18:2	14:0-12:0-18:2
18:2-16:0-18:1	18:0-18:0-18:1	12:0-14:0-16:0	18:2-18:2-16:1
18:1-16:0-16:0	8:0-8:0-10:0	16:0-17:0-18:1	10:0-18:2-16:0
18:1-16:0-18:0	10:0-16:0-18:0	18:0-18:1-18:0	18:0-20:1-18:1
18:1-18:1-18:1	12:0-18:1-18:2	16:0-22:5-18:0	10:0-16:0-14:0
16:0-18:1-18:1	16:0-16:0-10:0	18:1-16:1-18:3	16:0-15:0-18:0
18:1-16:0-12:0	16:0-18:0-18:1	18:1-20:1-18:1	14:0-18:2-14:0
18:1-14:0-18:1	18:0-12:0-18:1	14:0-12:0-18:0	12:0-10:0-18:0
18:1-16:0-14:0	16:0-16:0-16:1	16:0-18:2-16:1	10:0-16:0-10:0
18:1-16:0-16:1	18:0-16:0-16:1	14:0-18:0-16:0	14:0-16:0-18:3
18:2-18:1-18:1	16:0-16:1-18:0	18:2-18:0-18:3	18:1-18:1-17:0
18:1-14:0-16:0	16:0-16:0-14:0	18:1-20:1-18:2	12:0-16:0-18:3
18:1-18:2-18:1	18:1-17:0-18:1	12:0-12:0-18:0	18:1-20:0-18:1
18:2-16:0-16:0	12:0-18:0-18:1	10:0-18:2-18:1	15:0-16:0-18:0
18:2-14:0-18:1	12:0-12:0-8:0	14:0-18:0-14:0	16:0-15:0-16:0
18:1-12:0-18:1	16:0-12:0-18:0	16:0-18:3-16:0	20:1-18:1-18:2
18:1-16:0-10:0	16:1-16:1-18:1	16:0-18:1-20:0	4:0-18:1-4:0
12:0-18:1-18:1	16:0-16:0-20:5	17:0-16:0-18:2	10:0-12:0-18:0
18:1-16:1-18:1	10:0-14:0-18:1	16:0-15:0-18:2	16:0-16:1-18:1
18:2-16:1-18:1	18:1-16:0-20:1	12:0-16:1-18:1	16:0-17:0-16:1
16:1-18:1-18:1	12:0-12:0-18:1	16:0-16:0-17:0	14:0-14:0-10:0
16:0-18:1-18:1	18:1-15:0-18:2	16:0-20:5-16:0	14:0-18:2-16:0
18:1-16:0-12:0	16:0-15:0-18:1	18:1-18:1-20:1	14:0-10:0-16:0
18:1-14:0-18:1	16:0-16:0-18:3	12:0-18:1-12:0	14:0-14:0-16:1
18:1-16:0-14:0	12:0-12:0-12:0	14:1-18:0-16:1	17:1-17:0-17:1
18:1-16:0-16:1	18:2-12:0-18:2	16:1-18:2-18:1	16:0-14:0-18:3
18:2-18:1-18:1	12:0-18:2-18:1	14:0-18:1-16:1	18:1-16:0-24:1
18:1-14:0-16:0	10:0-18:0-16:0	16:0-20:4-18:1	10:0-12:0-16:0
18:1-18:2-18:1	18:1-18:3-18:1	14:0-14:0-8:0	18:0-18:0-18:0
18:2-16:0-16:0	18:0-18:0-18:2	18:0-18:2-18:3	10:0-8:0-10:0
18:2-14:0-18:1	15:0-16:0-18:1	18:0-20:4-18:1	14:0-18:1-16:0
18:1-12:0-18:1	12:0-12:0-14:0	14:0-16:0-22:5	18:1-16:0-22:1
18:1-16:0-10:0	16:0-18:1-16:1	17:1-14:0-18:1	18:1-18:1-20:2
12:0-18:1-18:1	14:0-18:1-18:0	10:0-18:1-18:2	18:1-14:1-18:2
18:1-16:1-18:1	16:0-12:0-18:2	12:0-16:0-16:1	16:0-20:4-16:0
18:2-16:1-18:1	12:0-18:0-14:0	18:1-12:0-18:3	14:0-14:0-14:0
16:1-18:1-18:1	12:0-18:0-18:2	14:0-18:1-18:3	17:0-16:0-18:0
18:0-16:0-18:2	16:0-18:1-22:6	16:0-12:0-16:0	17:0-12:0-18:1
18:1-18:1-18:0	12:0-12:0-18:2	10:0-18:0-14:0	18:1-17:1-18:2
16:0-16:0-18:0	8:0-14:0-12:0	14:0-16:1-18:1	18:0-16:0-20:1
18:1-16:0-18:3	14:0-16:1-18:0	18:1-18:1-17:1	14:0-18:3-16:0
18:2-18:2-18:1	12:0-18:1-16:0	18:1-10:0-18:1	17:1-17:1-17:0
18:1-16:0-22:6	14:0-14:0-18:0	10:0-18:0-18:2	16:1-16:1-16:0
16:0-18:2-18:1	10:0-10:0-8:0	14:0-14:0-16:0	17:0-18:0-18:2
16:0-14:0-18:1	18:0-18:0-16:0	18:1-18:1-16:0	18:0-10:0-18:2
16:0-18:2-18:0	18:0-14:0-16:1	14:0-12:0-16:0	16:0-16:1-18:3
18:2-18:1-18:2	12:0-18:1-18:0	17:1-16:0-18:1	16:1-18:3-18:1
18:0-18:2-18:1	12:0-14:0-18:2	18:0-18:3-18:2	12:0-15:0-18:1
16:0-18:1-18:2	12:0-14:0-12:0	10:0-18:0-12:0	17:0-14:0-18:1
12:0-16:0-18:2	16:1-14:0-22:6	18:0-16:1-14:1	16:0-18:1-17:0
18:0-14:0-18:1	18:1-17:0-18:2	10:0-12:0-18:2	10:0-10:0-10:0
18:1-18:0-18:2	8:0-10:0-8:0	18:1-20:2-18:1	6:0-14:0-12:0
18:0-18:1-18:2	16:0-16:1-18:2	8:0-12:0-10:0	16:1-18:0-17:1
18:1-18:1-16:1	18:1-18:1-10:0	18:1-18:2-20:1	14:0-16:0-14:0
18:1-12:0-18:2	12:0-12:0-16:0	8:0-16:0-18:1	12:0-17:0-18:1
14:0-16:0-18:2	16:0-17:0-18:2	12:0-16:1-18:0	14:0-18:3-18:2
16:0-12:0-18:1	10:0-12:0-18:1	18:0-17:0-18:1	16:0-16:0-17:1
16:0-16:0-16:0	18:2-16:1-18:2	12:0-18:1-18:3	12:0-18:3-18:1
16:0-22:6-18:1	14:0-14:0-12:0	18:0-14:1-16:1	12:0-10:0-16:0
18:2-18:2-18:0	18:0-18:2-18:0	14:0-12:0-14:0	15:0-16:0-16:1
16:0-18:1-18:0	16:1-14:0-18:1	13:0-13:0-13:0	17:1-18:0-18:1
18:0-16:0-18:0	16:0-14:0-16:0	16:0-16:0-4:0	16:0-20:4-17:1
18:1-16:1-18:2	17:1-18:0-22:6	18:1-18:0-20:4	14:0-16:0-20:5
16:0-14:0-18:2	16:0-20:1-18:1	16:0-24:1-22:6	18:0-14:0-17:1
16:0-14:0-18:0	16:0-16:1-16:0	12:0-18:1-16:1	17:0-16:0-16:1
17:1-16:0-22:6	18:1-16:0-20:4	12:0-12:0-10:0	16:0-16:0-8:0
16:0-18:0-18:2	16:0-18:1-20:4	18:1-10:0-18:2	16:0-18:4-18:1
16:0-16:0-22:5	18:0-12:0-18:2	14:0-20:5-16:0	16:1-14:0-18:2
10:0-16:0-18:2	14:0-18:1-14:0	15:0-18:1-18:2	16:1-16:1-18:2
16:1-18:1-18:2	12:0-16:0-14:0	17:0-16:0-20:5	18:1-18:1-20:0
18:1-18:1-12:0	14:0-14:0-18:2	16:0-14:0-20:5	14:0-16:1-18:2
16:1-16:1-18:0	18:1-14:0-18:3	16:1-12:0-18:1	24:1-16:0-22:6
18:1-18:1-18:3	12:0-18:2-16:0	18:1-17:1-18:1	18:0-20:3-18:2
18:2-16:0-18:3	22:6-22:6-22:6	16:0-15:0-20:5	15:0-14:0-18:1
16:1-16:0-18:1	16:0-18:1-20:1	10:0-16:0-12:0	10:0-18:2-18:0
16:1-18:0-16:1	18:0-15:0-18:1	17:1-16:0-18:2	16:1-12:0-18:2
12:0-16:0-18:0	14:0-18:0-18:1	8:0-14:0-10:0	16:0-17:0-18:0
14:0-18:1-18:2	20:0-16:0-18:1	14:0-18:3-18:1	18:1-22:1-18:2
12:0-14:0-18:1	12:0-14:0-18:0	18:2-14:0-18:3	15:0-18:1-18:0
18:1-18:0-18:1	15:0-16:0-18:2	18:1-14:0-20:4	16:1-16:0-18:3
16:0-16:0-12:0	12:0-18:0-16:1	16:0-16:0-15:0	16:0-17:1-18:0
18:2-14:0-18:2	16:1-18:1-16:1	22:0-16:0-18:1	14:0-10:0-18:0
16:0-18:1-18:3	17:0-22:6-18:1	10:0-14:0-18:0	20:0-18:2-18:1
12:0-18:0-16:0	12:0-18:0-12:0	18:2-18:2-12:0	12:0-10:0-18:2
18:1-15:0-18:1	16:0-16:0-20:4	24:0-16:0-18:1	16:0-20:1-18:0
14:0-14:0-18:1	12:0-18:2-14:0	12:0-18:2-18:0	12:0-18:3-18:2
18:1-18:1-14:0	16:0-18:3-18:1	17:0-18:1-18:2	16:0-17:0-16:0
17:0-16:0-18:1	16:0-18:0-16:1	18:2-18:2-10:0	12:0-18:2-16:1
14:0-16:0-18:0	10:0-14:0-18:2	10:0-14:0-12:0	18:1-20:3-18:1
8:0-8:0-8:0	12:0-16:0-12:0	14:0-16:1-22:6	18:1-19:0-22:6
16:1-16:0-18:2	16:0-14:0-22:5	10:0-14:0-16:0	18:0-16:0-17:1
18:2-18:0-18:2	18:0-12:0-16:1	16:1-17:0-16:1	16:0-17:1-18:2
8:0-16:0-12:0	18:0-20:3-18:1	16:0-18:0-20:1	12:0-14:0-18:3

**Table 3:** Most abundant triacylglycerol (TAG) species (>3 g 100 g^−1^ TAG) identified in human milk (*n* = 55).

TAG	Mean (g 100 g^−1^)	SD (r) (g 100 g^−1^)
18:1-16:0-18:1	13.90	2.09
18:2-16:0-18:1	5.95	1.53
18:1-16:0-16:0	5.68	1.64
18:1-16:0-18:0	5.15	1.36
18:1-18:1-18:1	3.64	1.49
16:0-18:1-18:1	2.88	1.00

**Table 4:** Median**,** standard deviation of intermediate reproducibility (SD(iR)), and relative standard deviation of intermediate reproducibility (CV(iR)) of OPO content in human milks. Results are expressed in mg 100 mL^−1^ of human milk.

Analyte	Median (HPLC-HRMS) *n* = 36	Median (UPLC-QQQ) *n* = 36	Bias Systematic Proportional	Median (UPLC-QQQ) *n* = 27	SD(iR)	CV(iR)
OPO	244	238	None (5% signifiance level)	170	2.4	14.0

SD(iR) and CV(iR)% refer to the ultra-performance liquid chromatography coupled with triple quadrupole mass spectrometry (UPLC-QQQ) method.

**Table 5:** OPO concentration in human milk samples at 30, 60 and 120 days *postpartum*. Results are expressed in mg 100 mL^−1^ of human milk.

Time (days)	Mean	SD (r)	Min	Max
30 (*n* = 48)	333	11.8	70	640
60 (*n* = 47)	337	17.0	90	940
120 (*n* = 48)	383	18.0	60	760

## Supplementary Materials

The following are available online.
