# Supplementary material for: Quantification of 1,3-olein-2-palmitin (OPO) and Palmitic Acid in sn-2 Position of Triacylglycerols in Human Milk by Liquid Chromatography Coupled with Mass Spectrometry
Source: Molecules. 2018 Dec 21;24(1):22. doi: 10.3390/molecules24010022 (PMC6337272; doi:10.3390/molecules24010022)
Supplement: Supplementary file 1 [file molecules-24-00022-s001.pdf]

Supplemental tables list triacylglycerol molecules considered for the analysis of human milk by high resolution mass spectrometry and the validation data.

Table S1. Median**,** standard deviation of intermediate reproducibility (SD(iR)), and relative standard deviation of intermediate reproducibility (CV(iR)) of triacylglycerol (TAG) species in the lowest calibration point defined as limit of quantification (LOQ). Results are expressed in mg 100mL^{-1}.

TAG	Median (n=7)	SD(iR)	CV(iR)
6:0-6:0-6:0	0.093	0.007	7.5
8:0-8:0-8:0	0.094	0.005	5.2
10:0-10:0-10:0	0.099	0.005	4.7
12:0-12:0-12:0	0.095	0.011	11.6
14:0-14:0-14:0	0.100	0.006	6.4
14:0-18:1-16:0	0.104	0.005	4.4
14:0-18:1-22:0	0.106	0.019	17.8
14:0-18:1-4:0	0.101	0.011	11.2
16:0-16:0-16:0	0.103	0.013	12.6
16:0-16:0-18:0	0.098	0.011	11.0
16:0-16:0-18:1	0.095	0.017	17.6
16:0-16:0-18:2	0.106	0.003	2.9
16:0-18:0-18:1	0.100	0.016	15.6
16:0-18:1-18:2	0.101	0.006	5.6
18:0-18:0-16:0	0.099	0.014	13.9
18:0-18:0-18:0	0.099	0.009	9.2
18:1-18:1-16:0	0.097	0.009	9.2
18:1-18:1-18:0	0.103	0.006	6.2
18:1-18:1-18:1	0.104	0.004	3.6
18:1-18:1-18:2	0.100	0.006	6.1
20:0-20:0-20:0	0.101	0.002	1.8

Table S2. Median, standard deviation of repeatability (SD(r)), relative standard deviation of repeatability (CV(r)), standard deviation of intermediate reproducibility (SD(iR)), and relative standard deviation of intermediate reproducibility (CV(iR)) of representative triacylglycerol regioisomers in human milk supplied from Leebio. Results are expressed in mg $100mL^{-1}$ of human milk.

TAG species	Median (n=14)	SD (r)	CV (r)	SD (iR)	CV (iR)
12:0-12:0-12:0	1.0	0.2	15.4	0.2	14.6
14:0-14:0-14:0	8.3	0.8	9.2	0.8	9.3
18:2-18:2-18:2	3.5	0.5	14.9	0.7	19.5
18:2-18:1-18:2	10.5	0.7	6.6	1.7	16.0
14:0-18:1-16:0	26.2	1.8	7.0	2.8	10.6
16:0-16:0-18:2	22.6	2.2	9.6	1.9	8.2
18:1-18:1-18:2	20.0	1.0	4.9	1.1	5.6
16:0-18:1-18:2	95.8	5.7	6.0	16.3	17.0
16:0-16:0-16:0	9.6	0.5	4.8	0.7	7.4
16:0-16:0-18:1	50.6	2.9	5.8	2.9	5.8
18:1-18:1-16:0	110.0	4.8	4.4	6.4	5.8
18:1-18:1-18:1	15.0	1.0	6.3	1.2	8.0
16:0-16:0-18:0	10.0	0.3	3.1	0.7	6.9
16:0-18:0-18:1	48.1	2.4	5.0	2.2	4.6
18:1-18:1-18:0	13.1	0.6	4.9	0.5	4.0
18:0-18:0-16:0	4.9	0.5	9.2	0.7	14.1
14:0-18:1-22:0	5.4	0.4	7.8	0.8	14.3
18:0-18:0-18:0	0.6	0.1	16.6	0.1	20.7

Table S3. Triacylglycerol molecules and their associated carbon numbers (CN), number of unsaturation (UN) and precursor ions selected for detection by HPLC-HRMS.

CN	UN	m/z $[M+NH4]^+$	CN	UN	m/z $[M+NH4]^+$	CN	UN	m/z $[M+NH4]^+$	CN	UN	m/z $[M+NH4]^+$
6	0	236.11286	40	3	706.59802	52	1	878.81712	63	8	1018.87972
7	0	250.12851	40	2	708.61367	52	0	880.83277	63	7	1020.89537
8	0	264.14416	40	1	710.62932	53	12	870.66062	63	6	1022.91102
9	0	278.15981	40	0	712.64497	53	11	872.67627	63	5	1024.92667
10	0	292.17546	41	9	708.51976	53	10	874.69192	63	4	1026.94232
11	0	306.19111	41	8	710.53541	53	9	876.70757	63	3	1028.95797
12	0	320.20676	41	7	712.55106	53	8	878.72322	63	2	1030.97362
13	0	334.22241	41	6	714.56671	53	7	880.73887	63	1	1032.98927
14	0	348.23806	41	5	716.58237	53	6	882.75452	63	0	1035.00492
15	0	362.25371	41	4	718.59802	53	5	884.77017	64	17	1014.75452
16	0	376.26936	41	3	720.61367	53	4	886.78582	64	16	1016.77017
17	0	390.28501	41	2	722.62932	53	3	888.80147	64	15	1018.78582
18	1	402.28501	41	1	724.64497	53	2	890.81712	64	14	1020.80147
18	0	404.30066	41	0	712.64497	53	1	892.83277	64	13	1022.81712
19	1	416.30066	42	10	720.51976	53	0	894.84842	64	12	1024.83277
20	0	418.31631	42	9	722.53541	54	12	884.67627	64	11	1026.84842
20	1	430.31631	42	8	724.55106	54	11	886.69192	64	10	1028.86407
20	0	432.33196	42	7	726.56671	54	10	888.70757	64	9	1030.87972
21	1	444.33196	42	6	728.58237	54	9	890.72322	64	8	1032.89537
21	0	446.34761	42	5	730.59802	54	8	892.73887	64	7	1034.91102

22	4	452.30066	42	4	732.61367	54	7	894.75452	64	6	1036.92667
22	3	454.31631	42	3	734.62932	54	6	896.77017	64	5	1038.94232
22	2	456.33196	42	2	736.64497	54	5	898.78582	64	4	1040.95797
22	1	458.34761	42	1	738.66062	54	4	900.80147	64	3	1042.97362
22	0	460.36326	42	0	740.67627	54	3	902.81712	64	2	1044.98927
23	4	466.31631	43	10	734.53541	54	2	904.83277	64	1	1047.00492
23	3	468.33196	43	9	736.55106	54	1	906.84842	64	0	1049.02057
23	2	470.34761	43	8	738.56671	54	0	908.86407	65	12	1038.84842
23	1	472.36326	43	7	740.58237	55	12	898.69192	65	11	1040.86407
23	0	474.37891	43	6	742.59802	55	11	900.70757	65	10	1042.87972
24	5	478.31631	43	5	744.61367	55	10	902.72322	65	9	1044.89537
24	4	480.33196	43	4	746.62932	55	9	904.73887	65	8	1046.91102
24	3	482.34761	43	3	748.64497	55	8	906.75452	65	7	1048.92667
24	2	484.36326	43	2	750.66062	55	7	908.77017	65	6	1050.94232
24	1	486.37891	43	1	752.67627	55	6	910.78582	65	5	1052.95797
24	0	488.39456	43	0	754.69192	55	5	912.80147	65	4	1054.97362
25	5	492.33196	44	10	748.55106	55	4	914.81712	65	3	1056.98927
25	4	494.34761	44	9	750.56671	55	3	916.83277	65	2	1059.00492
25	3	496.36326	44	8	752.58237	55	2	918.84842	65	1	1061.02057
25	2	498.37891	44	7	754.59802	55	1	920.86407	65	0	1063.03622
25	1	500.39456	44	6	756.61367	55	0	922.87972	66	18	1040.77017
25	0	502.41021	44	5	758.62932	56	13	910.69192	66	17	1042.78582
26	6	504.33196	44	4	760.64497	56	12	912.70757	66	16	1044.80147
26	5	506.34761	44	3	762.66062	56	11	914.72322	66	15	1046.81712
26	4	508.36326	44	2	764.67627	56	10	916.73887	66	14	1048.83277
26	3	510.37891	44	1	766.69192	56	9	918.75452	66	13	1050.84842
26	2	512.39456	44	0	768.70757	56	8	920.77017	66	12	1052.86407
26	1	514.41021	45	11	760.55106	56	7	922.78582	66	11	1054.87972
26	0	516.42586	45	10	762.56671	56	6	924.80147	66	10	1056.89537
27	6	518.34761	45	9	764.58237	56	5	926.81712	66	9	1058.91102
27	5	520.36326	45	8	766.59802	56	4	928.83277	66	8	1060.92667
27	4	522.37891	45	7	768.61367	56	3	930.84842	66	7	1062.94232
27	3	524.39456	45	6	770.62932	56	2	932.86407	66	6	1064.95797
27	2	526.41021	45	5	772.64497	56	1	934.87972	66	5	1066.97362
27	1	528.42586	45	4	774.66062	56	0	936.89537	66	4	1068.98927
27	0	530.44151	45	3	776.67627	57	12	926.72322	66	3	1071.00492
28	6	532.36326	45	2	778.69192	57	11	928.73887	66	2	1073.02057
28	5	534.37891	45	1	780.70757	57	10	930.75452	66	1	1075.03622
28	4	536.39456	45	0	782.72322	57	9	932.77017	66	0	1077.05187
28	3	538.41021	46	12	772.55106	57	8	934.78582	67	12	1066.87972
28	2	540.42586	46	11	774.56671	57	7	936.80147	67	11	1068.89537
28	1	542.44151	46	10	776.58237	57	6	938.81712	67	10	1070.91102
28	0	544.45716	46	9	778.59802	57	5	940.83277	67	9	1072.92667
29	6	546.37891	46	8	780.61367	57	4	942.84842	67	8	1074.94232
29	5	548.39456	46	7	782.62932	57	3	944.86407	67	7	1076.95797
29	4	550.41021	46	6	784.64497	57	2	946.87972	67	6	1078.97362

29	3	552.42586	46	5	786.66062	57	1	948.89537	67	5	1080.98927
29	2	554.44151	46	4	788.67627	57	0	950.91102	67	4	1083.00492
29	1	556.45716	46	3	790.69192	58	14	936.70757	67	3	1085.02057
29	0	558.47281	46	2	792.70757	58	13	938.72322	67	2	1087.03622
30	6	560.39456	46	1	794.72322	58	12	940.73887	67	1	1089.05187
30	5	562.41021	46	0	796.73887	58	11	942.75452	67	0	1091.06752
30	4	564.42586	47	12	786.56671	58	10	944.77017	68	13	1078.87972
30	3	566.44151	47	11	788.58237	58	9	946.78582	68	12	1080.89537
30	2	568.45716	47	10	790.59802	58	8	948.80147	68	11	1082.91102
30	1	570.47281	47	9	792.61367	58	7	950.81712	68	10	1084.92667
30	0	572.48846	47	8	794.62932	58	6	952.83277	68	9	1086.94232
31	6	574.41021	47	7	796.64497	58	5	954.84842	68	8	1088.95797
31	5	576.42586	47	6	798.66062	58	4	956.86407	68	7	1090.97362
31	4	578.44151	47	5	800.67627	58	3	958.87972	68	6	1092.98927
31	3	580.45716	47	4	802.69192	58	2	960.89537	68	5	1095.00492
31	2	582.47281	47	3	804.70757	58	1	962.91102	68	4	1097.02057
31	1	584.48846	47	2	806.72322	58	0	964.92667	68	3	1099.03622
31	0	586.50411	47	1	808.73887	59	15	962.72322	68	2	1101.05187
32	6	588.42586	47	0	810.75452	59	14	964.73887	68	1	1103.06752
32	5	590.44151	48	12	800.58237	59	13	952.73887	68	0	1105.08317
32	4	592.45716	48	11	802.59802	59	12	954.75452	69	12	1094.91102
32	3	594.47281	48	10	804.61367	59	11	956.77017	69	11	1096.92667
32	2	596.48846	48	9	806.62932	59	10	958.78582	69	10	1098.94232
32	1	598.50411	48	8	808.64497	59	9	960.80147	69	9	1100.95797
32	0	600.51976	48	7	810.66062	59	8	962.81712	69	8	1102.97362
33	6	602.44151	48	6	812.67627	59	7	964.83277	69	7	1104.98927
33	5	604.45716	48	5	814.69192	59	6	966.84842	69	6	1107.00492
33	4	606.47281	48	4	816.70757	59	5	968.86407	69	5	1109.02057
33	3	608.48846	48	3	818.72322	59	4	970.87972	69	4	1111.03622
33	2	610.50411	48	2	820.73887	59	3	972.89537	69	3	1113.05187
33	1	612.51976	48	1	822.75452	59	2	974.91102	69	2	1115.06752
33	0	614.53541	48	0	824.77017	59	1	976.92667	69	1	1117.08317
34	6	616.45716	49	12	814.59802	59	0	978.94232	69	0	1119.09882
34	5	618.47281	49	11	816.61367	60	13	966.75452	70	12	1108.92667
34	4	620.48846	49	10	818.62932	60	12	968.77017	70	11	1108.92667
34	3	622.50411	49	9	820.64497	60	11	970.78582	70	10	1112.95797
34	2	624.51976	49	8	822.66062	60	10	972.80147	70	9	1110.94232
34	1	626.53541	49	7	824.67627	60	9	974.81712	70	8	1116.98927
34	0	628.55106	49	6	826.69192	60	8	976.83277	70	7	1119.00492
35	6	630.47281	49	5	828.70757	60	7	978.84842	70	6	1121.02057
35	5	632.48846	49	4	830.72322	60	6	980.86407	70	5	1123.03622
35	4	634.50411	49	3	832.73887	60	5	982.87972	70	4	1125.05187
35	3	636.51976	49	2	834.75452	60	4	984.89537	70	3	1127.06752
35	2	638.53541	49	1	836.77017	60	3	986.91102	70	2	1129.08317
35	1	640.55106	49	0	838.78582	60	2	988.92667	70	1	1131.09882
35	0	642.56671	50	12	828.61367	60	1	990.94232	70	0	1133.11447

36	6	644.48846	50	11	830.62932	60	0	992.95797	71	7	1133.02057
36	5	646.50411	50	10	832.64497	61	13	980.77017	71	6	1135.03622
36	4	648.51976	50	9	834.66062	61	12	982.78582	71	5	1137.05187
36	3	650.53541	50	8	836.67627	61	11	984.80147	71	4	1139.06752
36	2	652.55106	50	7	838.69192	61	10	986.81712	71	3	1141.08317
36	1	654.56671	50	6	840.70757	61	9	988.83277	71	2	1143.09882
36	0	656.58237	50	5	842.72322	61	8	990.84842	71	1	1145.11447
37	6	658.50411	50	4	844.73887	61	7	992.86407	71	0	1147.13012
37	5	660.51976	50	3	846.75452	61	6	994.87972	72	7	1147.03622
37	4	662.53541	50	2	848.77017	61	5	996.89537	72	6	1149.05187
37	3	664.55106	50	1	850.78582	61	4	998.91102	72	5	1151.06752
37	2	666.56671	50	0	852.80147	61	3	1000.92667	72	4	1153.08317
37	1	668.58237	51	12	842.62932	61	2	1002.94232	72	3	1155.09882
37	0	670.59802	51	11	844.64497	61	1	1004.95797	72	2	1157.11447
39	8	668.48846	51	10	846.66062	61	0	1006.97362	72	1	1159.13012
38	7	670.50411	51	9	848.67627	62	16	988.73887	72	0	1161.14577
38	6	672.51976	51	8	850.69192	62	15	990.75452	73	6	1163.06752
38	5	674.53541	51	7	852.70757	62	14	992.77017	73	5	1165.08317
38	4	676.55106	51	6	854.72322	62	13	994.78582	73	4	1167.09882
38	3	678.56671	51	5	856.73887	62	12	996.80147	73	3	1169.11447
38	2	680.58237	51	4	858.75452	62	11	998.81712	73	2	1171.13012
38	1	682.59802	51	3	860.77017	62	10	1000.83277	73	1	1173.14577
38	0	684.61367	51	2	862.78582	62	9	1002.84842	73	0	1175.16142
39	8	682.50411	51	1	864.80147	62	8	1004.86407	74	6	1177.08317
39	7	684.51976	51	0	866.81712	62	7	1006.87972	74	5	1179.09882
39	6	686.53541	52	12	856.64497	62	6	1008.89537	74	4	1179.09882
39	5	688.55106	52	11	858.66062	62	5	1010.91102	74	3	1179.09882
39	4	690.56671	52	10	860.67627	62	4	1012.92667	74	2	1185.14577
39	3	692.58237	52	9	862.69192	62	3	1014.94232	74	1	1187.16142
39	2	694.59802	52	8	864.70757	62	2	1016.95797	74	0	1189.17707
39	1	696.61367	52	7	866.72322	62	1	1018.97362	75	1	1201.17707
39	0	698.62932	52	6	868.73887	62	0	1020.98927	75	0	1203.19272
40	9	694.50411	52	5	870.75452	63	12	1010.81712	76	1	1215.19272
40	8	696.51976	52	4	872.77017	63	11	1012.83277	76	0	1217.20837
40	7	698.53541	52	3	874.78582	63	10	1014.84842	77	0	1231.22402
40	6	700.55106	52	2	876.80147	63	9	1016.86407	78	0	1245.23967
40	5	702.56671									
40	4	704.58237									
